# Paediatric PARV4 infection in South Africa: relationship to age, maternal PARV4 status, and HIV infection

**DOI:** 10.1186/1471-2334-14-S2-P92

**Published:** 2014-05-23

**Authors:** Philippa Matthews, Colin Sharp, Amna Malik, Emily Adland, Pieter Jooste, Philip Goulder, Peter Simmonds, Paul Klenerman

**Affiliations:** 1 Nuffield Department of Medicine, University of Oxford, Oxford, OX1 3SY, UK; 2 Department of Infectious Diseases and Microbiology, Oxford University Hospitals, John Radcliffe Hospital, Oxford, OX3 9DU, UK; 3 Roslin Institute, University of Edinburgh, Easter Bush, Edinburgh, EH25 9RG, UK; 4 Department of Pediatrics, University of Oxford, Oxford, OX1 3SY, UK; 5 Paediatric Department, University of Free State, Kimberley Hospital, Kimberley, Northern Cape, South Africa; 6 NIHR Biomedical Research Center, John Radcliffe Hospital, Oxford OX3 9DU, UK

## Introduction

First identified in 2005, PARV4 is best characterized in Western cohorts where it is strongly associated with other blood borne viruses and occurs only in individuals with risk factors for parenteral infection (in particular, injecting drug users). However, studies in Africa have shown evidence of PARV4 in subjects with no clear risk factors for acquisition of blood borne viruses. The clinical significance of PARV4 remains uncertain, but there is growing interest in the role of coinfecting pathogens in shaping outcomes of HIV, and in chronic viral infections as determinants of immunological development in childhood.

## Materials and methods

We studied a cohort of HIV-infected mothers (n=32) and children (n=40; median age 28 months), and HIV-negative pediatric controls (n=24, median age 86 months) in Kimberley, South Africa. PARV4 serostatus was determined by ELISA for IgG to the PARV4 VP2 protein. HIV viral load was determined using the Abbott m2000 assay, and CD4 T cell count by flow cytometry.

## Results

In this cohort, 45/96 individuals (46.9%) were positive for PARV4 IgG, including 19/32 adults (59.4%) and 26/64 children (40.6%). The seroprevalence of PARV4 IgG increased with age, demonstrating acquisition throughout childhood, plateauing by age 6-10 years (Fig. 1; R2=0.75 by logistic regression). There was strong concordance between maternal and child infection, with the mother’s PARV4 status being a clear predictor of IgG in her offspring (p=0.006). HIV-negative individuals were more likely than HIV-positive subjects to be PARV4 IgG positive (p=0.03), although this relationship may be confounded by age. There was no relationship between PARV4 status and HIV viral load or CD4 T cell count in HIV-infected adults or children.

## Conclusions

The epidemiology of PARV4 in this cohort contrasts strikingly with that in Western countries: the prevalence of PARV4 IgG approached 50%, with evidence for ongoing transmission throughout the early years of life. The observed concordance within families is suggestive of maternal to child transmission. Future studies are planned, to investigate further the relationship between PARV4 status and outcomes of HIV infection.

